# Delphi consensus for the third-line treatment of metastatic colorectal cancer

**DOI:** 10.1007/s12094-023-03369-1

**Published:** 2024-02-27

**Authors:** Pilar García-Alfonso, Ruth Vera, Enrique Aranda, Elena Élez, Fernando Rivera

**Affiliations:** 1 Hospital General Universitario Gregorio Marañón, Instituto de Investigación Sanitaria (IiSGM), Universidad Complutense de Madrid, C/ Dr. Esquerdo, 46, 28007 Madrid, Spain; 2 Department of Oncology, University Hospital of Navarra, Instituto de Investigación Sanitaria de Navarra (IdISNA), Pamplona, Navarra Spain; 3 Department of Medical Oncology, Hospital Universitario Reina Sofía, Instituto Maimónides de Investigación Biomédica de Córdoba (IMIBIC), Universidad de Córdoba, Centro de Investigación Biomédica en Red de Cáncer (CIBERONC), Córdoba, Spain; 4 Vall d’Hebron University Hospital and Institute of Oncology (VHIO), Universitat Autònoma de Barcelona, Barcelona, Spain; 5 Hospital Universitario Marqués de Valdecilla, IDIVAL, Santander, Spain

**Keywords:** Delphi, Quality of life (QoL), Rechallenge, Regorafenib, Therapeutic sequence, Trifluridine/tipiracil (FTD/TPI)

## Abstract

**Purpose:**

The optimal drug regimen and sequence are still unknown for patients with metastatic colorectal cancer (mCRC) who are candidates for third-line (3L) or subsequent treatment. The aim of this study is to know the opinion of experts on the most appropriate treatment options for mCRC in 3L and to clarify certain clinical decisions in Spain.

**Methods:**

Using a modified Delphi method, a group of experts discussed the treatment in 3L of patients with mCRC and developed a questionnaire with 21 items divided into 5 sections.

**Results:**

After 2 rounds, the 67 panelists consulted agreed on 17 items (81%). They considered that the main objective of 3L is to equally increase survival and improve patients’ quality of life (QoL), but preferably the QoL. It was agreed that patients with mCRC in 3L prefer to receive active versus symptomatic treatment. Panelists considered trifluridine/tipiracil (FTD/TPI) to be the best oral treatment available to them in 3L. In patients with MSI-H or dMMR and *BRAF V600E*, the panelists mostly prefer targeted treatments. Panelists agreed the use of a therapeutic sequence that not only increases outcomes but also allows patients to be treated later. Finally, it was agreed that FTD/TPI has a mechanism of action that allows it to be used in patients refractory to previous treatment with 5-fluorouracil.

**Conclusion:**

The experts agreed with most of the proposed items on 3L treatment of mCRC, prioritizing therapeutic options that increase survival and preserve QoL, while facilitating the possibility that patients can continue to be treated later.

## Introduction

Colorectal cancer (CRC) is a public health problem. It is the third most common cancer in men and the second in women and represents the second leading cause of death by cancer worldwide [1]. In Spain, CRC represents the second most common tumor in both genders [2]. It is believed that in the next 30 years, the mortality rate for colon cancer will be reduced, but there will be an increase for rectal cancer [3].

Standard first- and second-line treatments are based on combinations of fluoropyrimidine plus oxaliplatin or irinotecan, associated with an anti-angiogenic monoclonal antibody or anti-epidermal growth factor receptor (EGFR), which is chosen based on the RAS mutational status, although the optimal sequence is still unknown [4, 5]. Although the prognosis for metastatic CRC (mCRC) is poor, these treatments have led to an increase in the survival of patients, reaching a median of 25 to 30 months [4, 5]. Nevertheless, there is a growing number of patients with mCRC being candidates for a third line (3L) or beyond, although the optimal drug regimen and sequence are still unknown in this setting.

Only the presence of RAS-activating mutations (KRAS/NRAS), present in 30% to 45% of mCRC, has proven to be a negative predictive biomarker of response to anti-EGFR [6]. Although no other validated predictive biomarkers have been described, there are other biomarkers of special interest, such as *BRAF* mutation (present in 8% to 10% of mCRC), human epidermal growth factor receptor 2 (HER2) amplification, high microsatellite instability (MSI-H), and ALK/ROS1/NTRK fusion/rearrangements (occur in 0.2–2.4% of mCRC) [7–11].

Taking in mind these biomarkers, several alternatives have been proposed in the 3L for patients who have not responded or are refractory to the previous lines: (1) sequential administration of the two oral drugs approved in this indication, trifluridine/tipiracil and regorafenib, which have shown a statistically significant benefit in progression-free survival (PFS) and overall survival (OS) with a different toxicity profile both in clinical trials and in real-life studies [12, 13]; (2) administration of an anti-EGFR, such as cetuximab or panitumumab, in treatment-naive patients with RAS wild type, which is increasingly rare because these drugs are usually indicated in first or second line [14]; (3) reuse drugs already administered that were discontinued owing to toxicity or progression (oxaliplatin, irinotecan, fluoropyrimidine, anti-angiogenics, and anti-EGFR [if RAS wild type]). In this case, high-quality evidence is limited, but this strategy is often used in routine clinical practice in the absence of alternative therapies, especially in patients with good performance status [15, 16]; (4) specific treatments for selected populations, such as dual inhibition of HER2 in HER2-positive CRC (i.e., tucatinib plus trastuzumab), immunotherapy in MSI-H, and intrahepatic therapies in limited disease or primarily located in the liver, although the main recommendation is to include patients in clinical trials [17].

Although there are different therapeutic options for the 3L treatment of mCRC, the optimal sequence of these therapies and the management of these patients in 3L are unknown. For this reason, the aim of this work is to develop a consensus to know the opinion of a panel of Spanish experts on the management of patients with mCRC in the 3L and to clarify certain points regarding clinical decisions in this setting.

## Methods

### Study design

The study used a modified Delphi method, a structured communication technique that allows a group of experts to gather opinions on a given complex or controversial topic for which there is insufficient evidence, or their knowledge is incomplete or uncertain [18, 19]. In addition, it allows the opinions of a group of experts to be explored and unified without the difficulties and inconveniences inherent to consensus methods based on face-to-face discussions, such as displacements or the biases of influence or non-confidential interaction.

The study was performed in three distinct phases from July 2021 to July 2022: (a) meeting of the scientific committee to raise the problem to be addressed and draft a statement list (July 2021); (b) two successive rounds of online surveys to obtain the opinion of a panel of experts about the items of the statement list (February–July 2022); and (c) analysis and discussion of the results to draw conclusion (July 2022).

### Participants

Three groups participated in the study: a scientific committee, a technical team, and a panel of experts. The scientific committee consisted of five oncologists involved in the treatment of patients with mCRC and provide care in public and private hospitals in Spain, whose role was to review the literature and draft a questionnaire with items regarding the treatment of mCRC in the 3L. The technical team, which directed and supervised the entire process, was responsible for the instrumental implementation of the method (search of the literature, distribution of the questionnaire to the panelists, analysis of the responses, and statistical interpretation of the consensus). Finally, the scientific committee chose the panel of experts, selected according to the type of center in which they work, their clinical experience, their experience in the management of patients with mCRC, their experience in the use of treatments for the disease, and according to the geographic region to which they belong, trying to achieve the maximum representation of all of Spain.

### Delphi questionnaire

Based on the discussions of the scientific committee, and according to the evidence found on the topic, the scientific committee developed a Delphi questionnaire consisting of 21 items grouped in 5 sections that included the most relevant controversies on the treatment of mCRC in the 3L: (1) 3L objectives (three items); (2) treatment options in 3L (seven items); (3) subgroup of patients (five items); (4) therapeutic sequences (four items); and (5) conditions of use of trifluridine/tipiracil (three items).

For the evaluation of the questionnaire, a single nine-point Likert-type ordinal scale was proposed, according to the model developed by the UCLA-RAND Corporation for the comparative evaluation and prioritization between different health-care options (minimum 1, complete disagreement; and maximum 9, complete agreement) [19]. This scale was structured in three groups according to the level of agreement–disagreement of the statement: from 1 to 3, interpreted as rejection or disagreement; from 4 to 6, interpreted as no agreement or disagreement; and from 7 to 9, interpreted as expression of agreement or support.

### Phases of Delphi consensus

Following the Delphi methodology procedure [20], the questionnaire was sent to the panel of experts to respond by showing their degree of agreement with the items. In the first round, the panelists responded to the questionnaire *online* and were offered the possibility of adding their opinion as an open text. The technical team evaluated and presented the results of the first round using bar graphs to facilitate comments and clarifications from each participant. In the second round, the panelists contrasted their personal opinion with that of the other participants and, if necessary, reconsidered their initial opinion on those items where consensus was not reached. The results of this second round were tabulated and presented descriptively. In a final meeting, the scientific committee discussed and interpreted the results.

### Analysis and interpretation of results

The median and interquartile range of the scores obtained for each item were used to analyze the data for both rounds. There was consensus when two-thirds or more of the respondents (≥ 66.7%) scored within the three-point range (1–3 or 7–9) that contained the median. The type of consensus reached on each item was determined by the score median. There was agreement if the median was ≥ 7 and there was disagreement if the median was ≤ 3. No consensus was considered when one-third or more of the panelists (≥ 33.3%) scored in the range of 1–3 and another third or more in the range of 7–9. When the median score fell between the range of 4 and 6, the items were considered uncertain to a representative majority of the group.

The size of the expert panel was determined as follows: with a minimum sample size of 50 experts, a 14% marginal error is obtained, for a confidence level of 95% and a heterogeneity of 50% in the sub-analyses by specialty. A total of 67 experts in the treatment of patients with mCRC were finally selected.

## Results

**Fig. 1:**
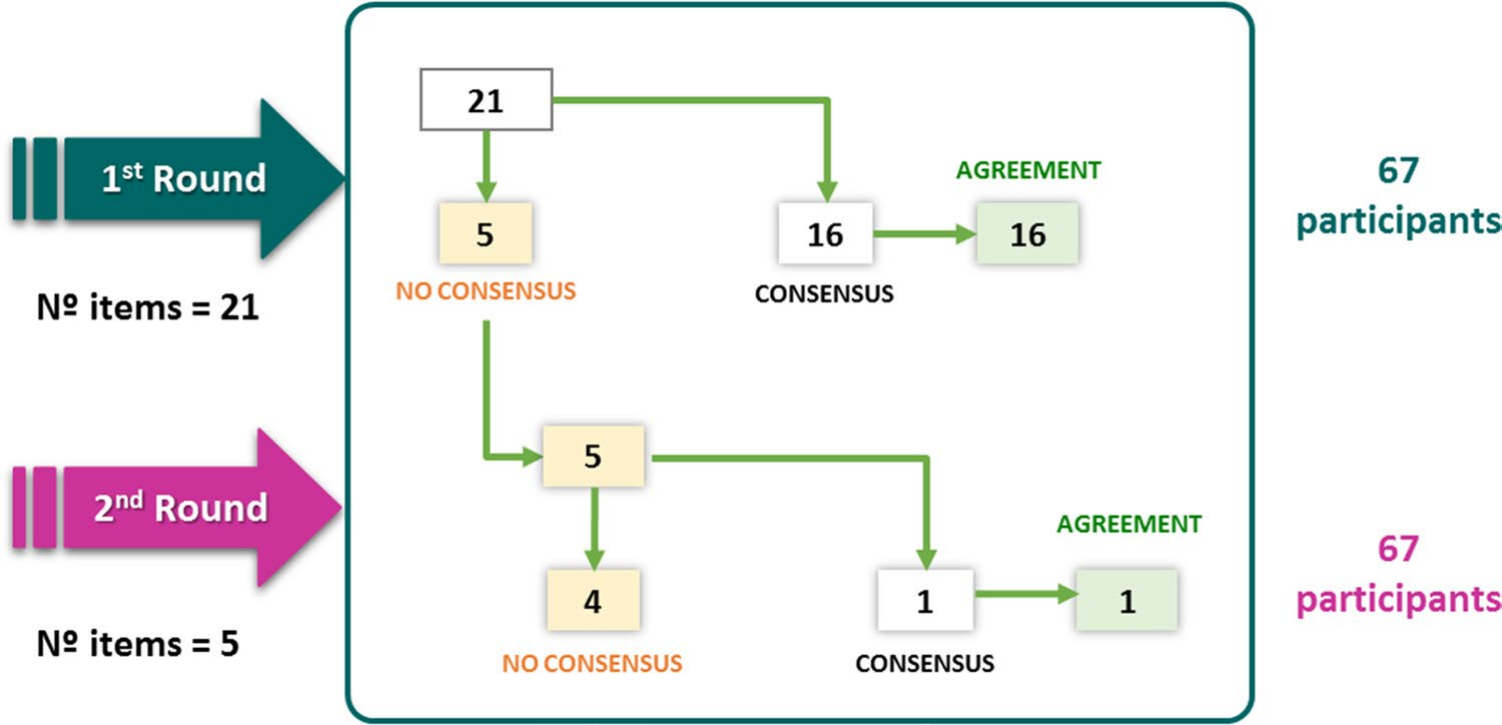
Main results of the Delphi consensus

The 67 experts consulted completed the 2 rounds of the Delphi consensus. Of the 21 items proposed in the first round, consensus was reached on 16 (76%), all of them in agreement. The five non-consensus items were sent to the panelists to be assessed in a second round. Of these, consensus was reached only in one item (in agreement) and four did not reach consensus. After the 2 rounds, with a total of 21 items, 17 reached consensus in agreement (81%) and 4 did not reach consensus (19%) (Fig. 1). Table 1 shows in detail the degree of agreement reached with each item after the two rounds.

**Table 1:** Results of Delphi consensus after two rounds

Items	Median (IQR)	% Agreement	Round with agreement
*3L objectives*			
1. The objective of 3L is to equally increase survival and improve the quality of life of patients	8 (6–9)	75	1st round
2. Although the objective of 3L is to equally increase survival and improve patients’ quality of life, survival is prioritized over patients’ quality of life	3 (2–5)	54	No consensus
3. Although the objective of 3L is to equally increase survival and improve patients’ quality of life, patients’ quality of life is prioritized over survival	8 (6–8)	70	2nd round
*Treatment options in the 3L*			
4. Patients with mCRC in 3L mostly prefer to receive active treatment rather than only symptomatic treatment	8 (8–9)	97	1st round
5. Of the oral treatments available in 3L, trifluridine/tipiracil is the drug that best combines, overall in most patients, an increase in survival, a manageable safety profile, and maintenance of functional status	8 (7–8)	90	1st round
6. Trifluridine/tipiracil is effective and safe in most patients with mCRC in 3L	7 (7–8)	75	1st round
7. Regorafenib is effective and safe in most patients with mCRC in 3L	5 (3–6)	46	No consensus
8. Before retreatment (rechallenge) with anti-EGFR, a liquid biopsy should always be performed	9 (8–9)	88	1st round
9. In patients with mCRC who have received anti-angiogenic treatment in 1L and 2L, maintenance of anti-angiogenic agent in 3L could benefit chemotherapy	5 (3–7)	34	No consensus
10. Clinical trials are one of the options for patients with mCRC in 3L if they meet the inclusion criteria	9 (8–9)	94	1st round
*Subgroups of patients*			
11. Trifluridine/tipiracil shows better results in OS and PFS in 3L in patients with mCRC and the following characteristics: ECOG PS 0–1, 1 or 2 metastatic sites, and time since first metastasis ≥ 18 months, and even better results in the same group of patients without liver metastases	8 (8–9)	94	1st round
12. Based on clinical experience in RAS/BRAF wild-type mCRC, retreatment (rechallenge) with anti-EGFR is an option to be assessed for patients who have PFS ≥ 4–6 months to anti-EGFR in 1L, an anti-EGFR-free treatment interval of at least 4 months, and remain wild type after liquid biopsy	8 (7–9)	88	1st round
13. Immunotherapy is indicated in patients with mCRC with MSI-H or with dMMR in 1L or later lines if they have not been previously treated with this option	9 (9–9)	99	1st round
14. If available, administration of cetuximab in 2L or 3L together with encorafenib may be an option in patients with mCRC and *BRAF V600E* mutation	9 (8–9)	97	1st round
*Therapeutic sequences*			
15. When establishing a therapeutic sequence beyond 2L, an option must be used that increases survival with good tolerance and ideally allows the patient to continue to be treated thereafter	8 (8–9)	96	1st round
16. To improve the survival of patients with mCRC, it is important to be able to administer as many lines of treatment as possible	8 (7–9)	94	1st round
17. The incorporation of oral drugs in 3L increases the survival of patients with mCRC	8 (7–9)	91	1st round
18. Trifluridine/tipiracil used in 3L preserves the functional status in a high percentage of patients, and therefore allows patients to be treated with other alternative therapies if necessary	8 (7–9)	91	1st round
*Conditions of use of trifluridine/tipiracil*			
19. In addition to dose reduction and other modifications of trifluridine–tipiracil therapy according to the degrees of neutropenia described in the data sheet, G-CSF was used sometimes in the case of neutropenia	7 (3–8)	54	No consensus
20. In the use of G-CSF after the administration of trifluridine/tipiracil on days 1–5 and 8–12 of each cycle, the G-CSF would be used on days 14–18 (from 1 to 5 doses during those days according to the patient’s need)	7 (6–8)	72	1st round
21. The mechanism of action of trifluridine/tipiracil, which is different from that of conventional fluoropyrimidine, provides evidence for its use in patients refractory to previous lines of 5-fluorouracil or who cannot receive it	8 (7–9)	90	1st round

1L: first line, 2L: second line, 3L: third line, *dMMR* mismatch repair deficient, *EGFR* epidermal growth factor receptor, *G-CSF* granulocyte colony-stimulating factor, *IQR* interquartile range, *mCRC* metastatic colorectal cancer, *MSI-H* microsatellite instability-high, *OS* overall survival, *PFS* progression-free survival

### Third-line objectives

Of the three items proposed, only one remained without consensus. Panelists agreed in the first round that the objective of 3L is to equally increase survival and improve the quality of life of patients (75% of agreement). In the second round, panelists agreed that although the objective of 3L is to equally increase survival and improve patients’ quality of life, patients’ quality of life is prioritized over survival (70%). The panelists differed on prioritizing survival over quality of life, and then the item did not reach consensus.

### Treatment options in third line

Of the seven items proposed, five reached consensus in agreement in the first round. The item with the highest degree of consensus stated that “patients with mCRC in third-line mostly prefer to receive active treatment rather than only symptomatic treatment” (97% of agreement). Other item with a high degree of agreement said that “clinical trials are one of the options for patients with mCRC in third-line if they meet the inclusion criteria” (94%). In addition, panelists agreed that “among the oral treatments available in third-line, trifluridine/tipiracil is the drug that best combines, overall in most patients, an increase in survival, a manageable safety profile, and maintenance of functional status” (90%).

The two items that remained without consensus stated that “regorafenib is effective and safe in most patients with mCRC in third-line”, and that “maintenance of the anti-angiogenic agent in third-line could benefit chemotherapy”.

### Subgroup of patients

The four items proposed were agreed with a high degree of consensus in the first round. One of them said that, “if available, administration of cetuximab in second- or third-line together with encorafenib may be an option in patients with mCRC and *BRAF V600E* mutation” (97% agreement). Another of the agreed items stated that “based on clinical experience in RAS/BRAF wild-type mCRC, retreatment (rechallenge) with anti-EGFR is an option to be assessed for patients who have PFS ≥ 4–6 months to anti-EGFR in first-line, an anti-EGFR-free treatment interval of at least 4 months, and remain wild-type after liquid biopsy” (88%).

### Therapeutic sequences

The four proposed items reached consensus in the agreement in the first round. The item with the highest degree of agreement stated that “when establishing a therapeutic sequence beyond second-line, an option must be used that increases survival with good tolerance and ideally allows the patient to continue to be treated thereafter” (94% agreement). Other items said that “trifluridine/tipiracil used in third-line preserves the functional status in a high percentage of patients and therefore allows patients to be treated with other alternative therapies if necessary” (91%).

### Conditions of use of trifluridine/tipiracil

Of the three items proposed, only one item did not reach consensus. This item stated that, “in addition to dose reduction and other modifications of trifluridine–tipiracil therapy according to the degrees of neutropenia described in the data sheet, I use G-CSF sometimes in case of neutropenia”. The item with the highest degree of agreement (reached in the first round) stated that “the mechanism of action of trifluridine/tipiracil, which is different from that of conventional fluoropyrimidines, provides evidence for its use in patients refractory to previous lines of 5-fluorouracil or who cannot receive it” (90% agreement).

## Discussion

Although the optimal pharmacologic regimen and sequence for patients with mCRC who are candidates for 3L or subsequent treatment are still unknown, the experts consulted showed a high degree of agreement with the proposed items (81%). However, there was no clearly defined position on 19% of the items, indicating there was no consensus. This shows that in certain specific cases of mCRC management in the 3L, the opinions and criteria of the experts on the same issues differ considerably.

Most of the panelists considered that the main objective of third-line is to equally increase survival and improve patients’ quality of life, but preferably the quality of life. Currently, all lines of treatment are required to increase overall survival, but prioritizing patient’s quality of life. Panelists argued that it would be equally valid to increase survival even at the cost of an “acceptable” deterioration in quality of life.

Regarding treatment options in the 3L, the option most agreed by the panelists was that patients preferred active treatment over symptomatic treatment. Although they considered trifluridine/tipiracil to be the best oral treatment available to patients with mCRC in 3L, there was no consensus on regorafenib. The panelists argued that although there is a group of patients who could benefit from regorafenib, this drug has adverse events that often require dose adjustments [21]. In relation to the maintenance of anti-angiogenics in 3L to improve chemotherapy, despite being feasible due to their mechanism of action, the panelists considered that more clinical evidence is still needed [15]. Even some commented that it is not necessary to continue using the anti-angiogenic drug when there are alternatives such as regorafenib. At the time of this Delphi consensus, the SUNLIGHT study had not been published and was, therefore, not part of the questionnaire. The results of this study support that trifluridine/tipiracil plus bevacizumab may represent a new standard of care for the treatment of patients with refractory mCRC who have progressed after two lines of therapy [22]. Even more, the ESMO living guidelines for mCRC include this therapeutic combination for the 3L and beyond [23]. However, even if the study and ESMO recommendations had been published prior to our Delphi consensus, this combination still does not have a reimbursement price in Spain and cannot be used, so health-care professionals would continue to administer the 3L with the available options, options that would remain valid for those who are not candidates for trifluridine/tipiracil plus bevacizumab combination therapy.

In a specific group of patients, panelists mostly prefer targeted treatments, such as cetuximab together with encorafenib in patients with *BRAF V600E*, and immunotherapy in patients with MSI-H or dMMR. Clinical evidence with these therapeutic strategies seems to be valid to the experts [14, 24–27]. Even so, they also consider that trifluridine/tipiracil offers better outcomes in patients with ECOG PS 0–1, one or two metastatic sites, time from first metastasis  ≥ 18 months, and those without liver metastases. In addition, they considered the option of rechallenge with anti-EGFR in patients with RAS/BRAF wild-type mCRC, PFS ≥ 4–6 months to this therapy in first line, with an anti-EGFR-free interval of at least 4 months, and remain wild type after liquid biopsy [28].

Panelists agreed the use of a therapeutic sequence that not only increases outcomes but also allows patients to be treated later. However, it is interesting to note one of the most widely agreed items, the use of as many lines as possible to improve patient survival [29].

It has been described that the mechanism of action of trifluridine/tipiracil is different from that of the other fluoropyrimidine. This allows this drug to be used in those patients refractory to previous treatment with 5-fluorouracil or in whom this treatment cannot be administered [30, 31]. Panelists agreed with this information. Despite the scarce evidence (LONGBOARD trial, Clinicaltrials.gov ID: NCT04166604), opinions on the use of G-CSF after trifluridine/tipiracil administration are noteworthy. Some panelists commented that although there is no study to support it, the use of G-CSF is common due to the experience acquired with other chemotherapies, to avoid delays and to obtain the maximum benefit from the molecule. Other panelists explained that neutropenia caused by trifluridine/tipiracil is not severe and can be managed with delay in treatment by maintaining the dose without the need to use G-CSF.

Despite the benefits of this methodology, this work has some limitations. The selection of panelists was neither systematic nor randomized; recruitment was based on their clinical expertise in mCRC management. Panelists were recruited from different Spanish regions, although not all regions were represented. Finally, the results may have been influenced by ambiguity in the phrasing of some of the statements. In addition, panelists received honoraria from the sponsor. The fact that the survey was online had the advantage of anonymity, but it could lead to erroneous interpretations of the statements by the panelists, which could influence the result.

## Conclusion

This Delphi consensus is of particular relevance and highlights expert opinions on the treatment of patients with mCRC in 3L. The experts agreed with most of the proposed items on 3L treatment of mCRC, prioritizing therapeutic options that increase survival and preserve quality of life, while facilitating the possibility that patients can continue to be treated later. The lack of consensus observed in some items suggests the need to improve knowledge about them.

## Data Availability

Not applicable.

## Appendix

See below Table 2.

**Table 2:** Panel of experts participating in the Delphi consensus

	Expert	Hospital	City
1	Rosario Vidal Tocino	C.A. Universitario de Salamanca	Salamanca
2	Margarita Reboredo López	CHU A Coruña	A Coruña
3	Nieves Purificación Martínez Lago	CHUAC	A Coruña
4	Javier Sastre Valera	Clinico San Carlos	Madrid
5	Beatriz García Paredes	Clínico San Carlos	Madrid
6	Sonia María Candamio Folgar	Clínico Universitario de Santiago de Compostela	Santiago de Compostela
7	Carmen Castañón López	Complejo Asistencial Universitario de León	León
8	Isabel Ruiz Martín	Complejo Asistencial Universitario de Palencia	Palencia
9	Mercedes Salgado Fernández	Complejo Hospitalario Universitario de Ourense	Ourense
10	Antía Cousillas Castiñeiras	Complejo Hospitalario Universitario de Pontevedra	Pontevedra
11	Ana Fernández Montes	Complexo Hospitalario Universitario de Ourense	Ourense
12	María José Safont Aguilera	Consorcio Hospital General Universitario de Valencia	Valencia
13	Javier Gallego Plazas	General Universitario de Elche	Elche
14	Juan Carlos Cámara Vicario	H. Universitario Fundación Alcorcón	Alcorcón
15	Carlos López López	H.U. Marqués de Valdecilla	Santander
16	M. Luisa Limón Mirón	H.Virgen del Rocío	Sevilla
17	María Luisa Gonzálvez Cascales	HCUVA	Murcia
18	Susana Roselló Keranen	Hospital Clínico Universitario de Valencia	Valencia
19	María Francisca Vázquez Rivera	Hospital Clínico Universitario Santiago de Compostela	Santiago de Compostela
20	David Gutiérrez Abad	Hospital de Fuenlabrada	Fuenlabrada
21	David Páez	Hospital de la Santa Creu i Sant Pau	Barcelona
22	Juana María Cano Cano	Hospital General de Ciudad Real	Ciudad Real
23	Ana López Alfonso	Hospital Infanta Leonor	Madrid
24	María Aránzazu Fernández Orgiler	Hospital Marina Baixa	Villajoyosa
25	Eduardo Polo Marqués	Hospital Miguel Servet Zaragoza	Zaragoza
26	Alberto Carmona Bayonas	Hospital Morales Meseguer	Murcia
27	Rosa Querol Niñerola	Hospital Parc Taulí	Sabadell
28	Jorge Molina Saera	Hospital Provincial de Castellón	Castellón
29	Yolanda López Mateos	Hospital Provincial de Zamora	Zamora
30.	Patricia Ramírez Daffós	Hospital Puerta del Mar	Cádiz
31	Carles Bosch Roig	Hospital Universitari Dr. Peset	Valencia
32	Cristina Grávalos Castro	Hospital Universitario 12 de Octubre	Madrid
33	Rocío García Carbonero	Hospital Universitario 12 de Octubre	Madrid
34	Bartomeu Massutí Sureda	Hospital Universitario Alicante Dr. Balmis Isabial	Alicante
35	Jose Pablo Berros Fombella	Hospital Universitario Central de Asturias	Oviedo
36	Beatriz González Astorga	Hospital Universitario Clínico San Cecilio	Granada
37	Juan de la Cámara Gómez	Hospital Universitario de A Coruña	A Coruña
38	Ana María López Muñoz	Hospital Universitario de Burgos	Burgos
39	Mª Rosario Dueñas García	Hospital Universitario de Jaén	Jaén
40	Encarnación Jiménez Orozco	Hospital Universitario de Jerez	Jerez de La Frontera
41	Jesús García-Foncillas López	Hospital Universitario Fundación Jiménez Díaz	Madrid
42	Enrique Casado Sáenz	Hospital Universitario Infanta Sofía	San Sebastián de los Reyes
43	Nuria Rodríguez Salas	Hospital Universitario La Paz	Madrid
44	María Ruth Afonso Gómez	Hospital Universitario Nuestra Señora de Candelaria	Santa Cruz de Tenerife
45	Sandra Rubiales Trujillano	Hospital Universitario Puerto Real	Puerto Real
46	María José Ortiz Morales	Hospital Universitario Reina Sofía	Córdoba
47	Juan José Reina Zoilo	Hospital Universitario Virgen Macarena	Sevilla
48	María Dolores Mediano Rambla	Hospital Universitario Virgen Macarena	Sevilla
49	Jorge Aparicio Urtasun	Hospital Universitario y Politécnico La Fe	Valencia
50	José Antonio Santiago Crespo	Hospital Virgen de la Luz	Cuenca
51	Fernando Garicano Goldaraz	HU Galdakao	Bilbao
52	Ana Ruiz Casado	HU Puerta de Hierro Majadahonda	Majadahonda
53	Paula Jiménez Fonseca	HUCA	Oviedo
54	Marcos Melián Sosa	Instituto Valenciano de Oncología	Valencia
55	Matilde Bolaños Naranjo	Juan Ramón Jiménez	Huelva
56	Rafael Morales Chamorro	La Mancha Centro	Alcázar de San Juan
57	Ismael Ghanem Cañete	La Paz	Madrid
58	Jaime Feliu Batlle	La Paz	Las Rozas
59	Vicente Alonso Orduña	Miguel Servet	Zaragoza
60	Maialen Barrero Iñiguez	Onkologikoa/Hospital Donostia	Donostia
61	Reyes Ferreiro Monteagudo	Ramón y Cajal	Madrid
62	Jorge Muñoz Luengo	San Pedro de Alcántara	Cáceres
63.	Teresa Fernández Rodríguez	Son Llatzer	Palma de Mallorca
64	Antonieta Salud Salvia	Universitario Arnau de Vilnova	Lleida
65	Marta Llanos Muñoz	Universitario de Canarias	La Laguna
66	Encarna González Flores	Virgen de las Nieves	Granada
67	Manuel Ayerbes Valladares	Virgen del Rocío	Sevilla
